# Tumor angiogenesis at baseline identified by ^18^F-Alfatide II PET/CT may predict survival among patients with locally advanced non-small cell lung cancer treated with concurrent chemoradiotherapy

**DOI:** 10.1186/s12967-022-03256-3

**Published:** 2022-02-02

**Authors:** Yuchun Wei, Xueting Qin, Xiaoli Liu, Jinsong Zheng, Xiaohui Luan, Yue Zhou, Jinming Yu, Shuanghu Yuan

**Affiliations:** 1 Cheeloo College of Medicine, Shandong University, Jinan, China; 2 Department of Radiology, Shandong Cancer Hospital and Institute, Shandong First Medical University and Shandong Academy of Medical Sciences, Jinan, 250117 Shandong China; 3 Department of PET/CT Center, Shandong Cancer Hospital and Institute, Shandong First Medical University and Shandong Academy of Medical Sciences, Jinan, Shandong China; 4 Department of Radiology, Dezhou People’s Hospital, Dezhou, Shandong China; 5 Department of Oncology, Shanghe People’s Hospital, Jinan, Shandong China

**Keywords:** ^18^F-alfatide, PET/CT, Non-small cell lung cancer, Chemoradiotherapy

## Abstract

**Background:**

The study investigated the predictive value of tumor angiogenesis observed by ^18^F-ALF-NOTA-PRGD2 II (denoted as ^18^F-Alfatide II) positron emission tomography (PET)/computed tomography (CT) before concurrent chemoradiotherapy (CCRT) for treatment response and survival among patients with locally advanced non-small cell lung cancer (LA-NSCLC).

**Methods:**

Patients with unresectable stage IIIA or IIIB NSCLC (AJCC Cancer Staging 7th Edition) who received CCRT were included in this prospective study. All patients had undergone ^18^F-Alfatide PET/CT scanning before CCRT, and analyzed parameters included maximum uptake values (SUV_max_) of primary tumor (SUV_P_) and metastatic lymph nodes (SUV_LN_) and mean uptake value of blood pool (SUV_blood_). Tumor-to-background ratios (TBRs) and changes in tumor diameter before and after CCRT (ΔD) were calculated. The ratios of SUV_P_ to SUV_blood_, SUV_LN_ to SUV_blood_, and SUV_P_ to SUV_LN_ were denoted as TBR_P_, TBR_LN_, and T/LN. Short-term treatment response, progression-free survival (PFS), and overall survival (OS) were evaluated.

**Results:**

Of 38 enrolled patients, 28 completed CCRT. SUV_P_, SUV_LN_, TBR_P_, TBR_LN_ and T/LN showed significant correlation with PFS (all *P* < 0.05). SUV_P_ was negatively correlated with OS (*P* = 0.005). SUV_P_ and TBR_P_ were higher in non-responders than in responders (6.55 ± 2.74 vs. 4.61 ± 1.94, *P* = 0.039; 10.49 ± 7.58 vs. 7.73 ± 6.09, *P* = 0.023). ΔD was significantly greater in responders (2.78 ± 1.37) than in non-responders (-0.16 ± 1.33, *P* < 0.001). Exploratory receiver operating characteristic curve analysis identified TBR_P_ (area under the curve [AUC] = 0.764, *P* = 0.018), with a cutoff value of 6.52, as the only parameter significantly predictive of the response to CCRT, with sensitivity, specificity, and accuracy values of 71.43%, 78.57%, and 75.00%, respectively. ROC curve analysis also identified SUV_P_ (AUC = 0.942, *P* < 0.001, cutoff value 4.64) and TBR_P_ (AUC = 0.895, *P* = 0.001, cutoff value 4.95) as predictive of OS with high sensitivity (84.21%, 93.75%), specificity (100.00%, 66.67%), and accuracy (89.29%, 82.14%).

**Conclusions:**

Evaluation of tumor angiogenesis by ^18^F-Alfatide II at baseline may be useful in predicting the short-term response to CCRT as well as PFS and OS in patients with LA-NSCLC.

## Background

Locally advanced non-small cell lung cancer (LA-NSCLC) refers to stage III NSCLC, which is not suitable for treatment by surgical resection in the late stage of local foci [1]. These patients have regional lymph node invasion but no distant organ metastasis. Concurrent chemoradiotherapy (CCRT) is the standard treatment for LA-NSCLC [2, 3]. Vascular networks are derived through formation of new blood vessels via angiogenesis, which contributes to the vascular heterogeneity in and among tumors [4]. The abnormal structure of the tumor neovascularization bed is not conducive to drug delivery into solid tumors and also the main reason for the formation of the tumor hypoxic environment, which results in poor efficacy of chemotherapy as well as radiotherapy resistance [5]. Functional molecular imaging by positron emission tomography (PET) can be used to detect angiogenesis within a whole tumor.

PET/CT with a tracer based on the peptide arginine-glycine-aspartic acid peptide (Arg-Gly-Asp, commonly referred to as RGD) provides accurate imaging of integrin αvβ3 expression [6, 7] and thus is widely used to evaluate tumor angiogenesis and regarded as an ideal diagnostic tool for detecting tumors. An ^18^F-labeled PET imaging probe for RGD (^18^F-ALF-NOTA-PRGD2 II, denoted as ^18^F-Alfatide II) represents a potentially feasible imaging tool for assessing tumor angiogenesis [8–10]. Studies of ^18^F-Alfatide II have proven its safety and effectiveness for evaluating angiogenesis in NSCLC [11] with clarity and desirable imaging contrast, and phase III clinical trials are under way [12]. Small sample, prospective studies have suggested that RGD PET results have the ability to predict the short-term outcomes of antineoplastic therapy [13]. To date, a few studies have reported the use of RGD PET to predict short-term outcomes [14] and PFS [15] in cancer patients, but there are no related research reports on the ability to RGD PET results to predict long-term survival, especially in patients with locally advanced non-small cell lung cancer (LA-NSCLC).

In this study, we analyzed the standard uptake values (SUVs) of ^18^F-Alfatide II on PET/computed tomography (CT) at baseline and explored the predictive value of these and related parameters for short-term treatment outcome, progression-free survival (PFS) and overall survival (OS) in LA-NSCLC patients treated with CCRT.

## Methods

### Patients

Between June 10, 2015, and Dec 28, 2016, a total of 38 patients with unresectable stage III NSCLC were enrolled in the study. This prospective study was approved by the local ethics committee of Shandong Cancer Hospital and Institute, and each patient gave written and informed consent before inclusion in the study. All patients were treated in Shandong Cancer Hospital and met the following criteria: (1) NSCLC diagnosed by histological and imaging examination such as CT or ^18^F-fluorodeoxyglucose (FDG) PET/CT; (2) Eastern Cooperative Oncology Group (ECOG) score ≤ 1; (3) clearly measurable metastatic lymph nodes and primary tumors (RECIST); and (4) age > 18 years. All patients were ready to undergo ^18^F-Alfatide II PET/CT before chemotherapy or radiotherapy.

### Radiotracer preparation

A simple lyophilized kit for labeling with PRGD2 peptide was purchased from the Jiangsu Institute of Nuclear Medicine, and the synthesis process was carried out as described in a previous study [16]. The radiochemical purity of the ^18^F-alfatide II exceeded 99%, and its specific radioactivity exceeded 37 GBq (1,000 mCi)/μmol.

### PET/CT scanning

Patients were given an intravenous injection of 4.81 MBq/kg (0.12 mCi/kg) ^18^F-alfatide II and rested for about 60 min. Patients were not asked to fast or to confirm blood glucose levels. Scanning was performed with an integrated in-line PET/CT system (GEMINI TF Big Bore; Philips Healthcare). PET images were obtained from the head to the thigh, and the spiral CT component was performed with an X-ray tube voltage peak of 120 kV, 300 mAs. A full-ring dedicated PET scan of the same axial range followed. The patients continued normal shallow respiration during image acquisition. The images were attenuation-corrected with the transmission data from CT. The attenuation-corrected PET images, CT images, and fused PET/CT images, displayed as coronal, sagittal, and trans axial slices, were viewed on a MEDEX workstation (Beijing, China).

### Image analysis

Two experienced nuclear medicine physicians assessed the ^18^F-alfatide II PET/CT images visually, referring to PET fusion and CT images, until consensuses were reached. Acquired ^18^F-alfatide II PET/CT data were transferred to the workstation in the DICOM format. The radiotracer concentration in the region of interest (ROI) was normalized to the injected dose per kilogram of each patient’s body weight to derive the standardized uptake values (SUVs). The SUVs were calculated according to the following formula: [measured activity concentration (Bq/ml) × body weight (g)]/injected activity (Bq).

PET/CT parameters were generated using a vendor-provided automated contouring program. Tumor tracer uptake was quantified according to the maximum standard uptake value (SUVmax) at 1 h after injection. For calculation of the SUVmax of the primary tumor (SUV_P_) and metastatic lymph nodes (SUV_LN_), circular regions of interest (ROIs) were drawn around the tumor lesions with a focally increased uptake in transaxial slices and automatically adapted to a 3-dimensional volume with a 30% isocontour. In addition, the maximal activity of 1 cm^3^ within the aortic arches was measured as mean SUV for aortic arches and denoted as SUV_blood_. The SUV ratio for primary tumor to aortic arch was equal to SUV_P_/ SUV_blood_; that for metastatic lymph node to aortic arch was equal to SUV_LN_/ SUV_blood_; and that for primary tumor to metastatic lymph node was equal to SUV_P_/ SUV_LN_. ΔD refers to the maximum diameter of the tumor before treatment minus the diameter of the tumor within 1 month after CCRT treatment.

### Chemoradiotherapy

Patients were treated with two cycles of chemotherapy, followed by CCRT. Two additional cycles of chemotherapy were needed every 3 weeks after radiotherapy. An intensity-modulated radiotherapy technique (IMRT) or three-dimensional conformal RT (3D-CRT) was delivered to all patients with megavoltage equipment (6 MV). Radiotherapy was given as the conventionally fractionated regimen, 180–200 cGy for 5 days per week, and the total dose administered to patients ranged from 5040–6600 cGy (median dose, 6000 cGy). The chemotherapy administered was the cisplatin/docetaxel or cisplatin/pemetrexed regimen.

### Response evaluation and survival assessments

Short-term outcome was assessed at 4 weeks after CCRT according to the revised RECIST criteria (v.1.1). The size change and curative effect of tumor lesions were determined by enhanced CT examination (scanning slice thickness 3 mm), and using at least one size that could be accurately measured, a tumor lesion diameter ≥ 10 mm and a metastatic lymph node short axis diameter ≥ 15 mm were taken as measurable lesions. The baseline data of the target area were recorded individually and compared with the measured data after treatment.

According to the RECIST criteria, responders included patients with an outcome of complete response (CR) or partial response (PR), and patients who had an outcome of stable disease (SD) or progressive disease (PD) were classified as the non-responders.

PFS and OS were assessed for all NSCLC patients according to the RECIST criteria. OS and PFS were estimated from initiation of chemotherapy to death (for OS) and to progression or death (for PFS). Patients were followed up by enhanced CT every 6 weeks during treatment, every 2 months in the first year after treatment, and every 6 months from the second year after treatment.

### Statistical analysis

Statistical analysis was performed using IBM SPSS Statistics for Windows version 20.0 (IBM, Armonk, NY, USA). Quantitative data for SUV_P_, SUV_LN_, T/LN, TBR_P_ and TBR_LN_ were expressed as mean ± standard deviation or median (range). Two-sample *t* test and Wilcoxon rank-sum tests were used to compare the PET/CT parameters between responders and non-responders. PET/CT parameters were tested by logistic regression analyses to identify the relationships between these variables and short-term outcomes or survival. Receiver-operating characteristic (ROC) curve analysis was used to determine the thresholds with the maximum Youden index as well as the predictive accuracy of ^18^F-Alfatide II PET/CT parameters for treatment response and survival. Both PFS and OS were assessed by Kaplan–Meier analysis. All tests were two-sided, and *P* < 0.05 was considered statistically significant.

## Result

### Patient characteristics

A total of 38 patients with pathologically proven stage III NSCLC were enrolled in the study, and of these, 28 completed CCRT and were included in the final analysis (Table 1). As of March 01, 2021, the median follow-up was 36.50 months (range, 9.00–72.50 months). The median PFS was 17 months (95% CI, 7.89 ~ 26.12 months), and the median OS was 36 months (95% CI, 11.24 ~ 60.76 months). The 1-, 3-, and 5-year survival rates were 89.29%, 53.57% and 21.43%, respectively.

**Table 1:** Clinicopathological features of the patients with locally advanced NSCLC

Characteristics	Number of cases (%)
Age	60 ± 9.31
> 60	14 (50.00)
≤ 60	14 (50.00)
Gender	
Male	24 (85.71)
Female	4 (14.29)
Stage	
IIIA	13 (46.43)
IIIB	15 (53.57)
Pathological type	
Adenocarcinoma	11 (39.29)
Squamous cell carcinoma	17 (60.71)
Short-term outcome (RECIST)	
Complete response	1 (3.57)
Partial response	14 (50.00)
Stable disease	8 (28.57)
Progressive disease	5 (17.86)
PFS	17 (range 7.89 ~ 26.12) months
OS	36 (range 11.24 ~ 60.76) months
1 year survival rate	25 (89.29)
3 years survival rate	15 (53.57)
5 years survival rate	6 (21.43)

### Correlations between PET parameters and survival

The results of the correlation analyses for significant relationships between ^18^F-Alfatide II PET/CT semi-quantitative parameters and the PFS and OS of LA-NSCLC patients treated with CCRT are given in Table 2. SUV_P_, SUV_LN_, TBR_P_, TBR_LN_ and T/LN were correlated significantly with PFS, and among them, SUV_P_, TBR_LN_, T/LN and ΔD were negatively correlated with PFS. Only SUV_P_ was negatively correlated with OS (*P* = 0.005). Statistically significant correlations were not observed for the other parameters.

**Table 2:** Results of correlation and regression analysis between variables with PFS and OS

	PFS^a^				OS^b^			
	t	Sig	95.0% CI		t	Sig	95.0% CI	
			Lower bound	Upeper bound			Lower bound	Upeper bound
SUV_P_	− 3.802	0.001	− 25.779	− 7.582	− 3.145	0.005	− 22.410	− 4.601
SUV_LN_	3.509	0.002	8.531	33.196	1.662	0.111	− 2.395	21.745
TBR_P_	3.239	0.004	3.874	17.662	1.641	0.115	− 1.409	12.086
TBR_LN_	− 3.809	0.001	− 30.401	− 8.967	− 1.783	0.088	− 19.504	1.473
T/LN	− 3.023	0.006	− 27.602	− 5.140	− 1.465	0.157	− 18.756	3.228
△D	1.929	0.065	− 0.002	0.077	1.856	0.075	− 0.003	0.066

*△D* changes of tumor diameter before and after treatment

^a^Dependent variable, PFS

^b^Dependent variable, OS

### Predictive value of PET parameters for short-term treatment outcome

**Fig. 1:**
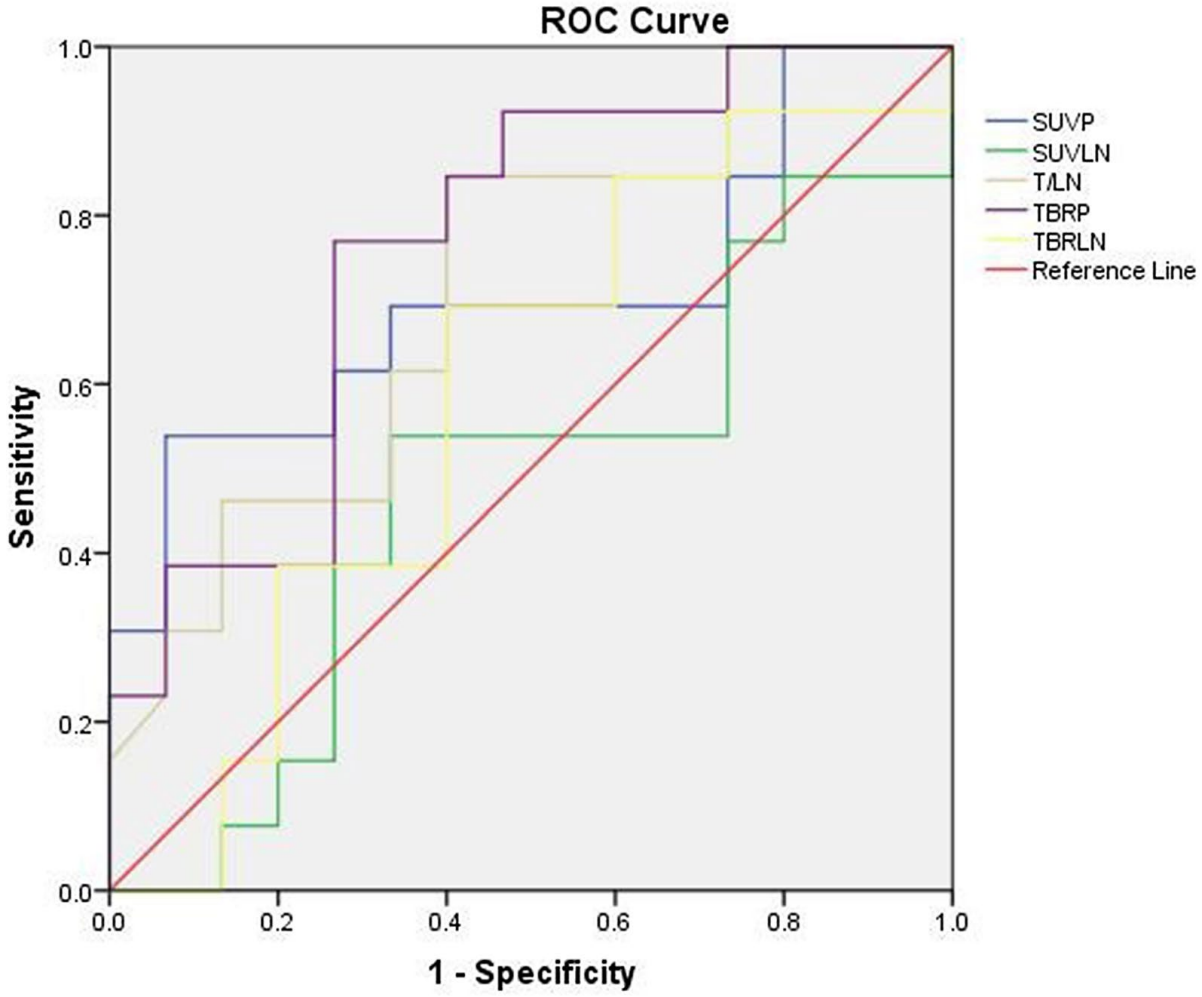
Two representatives ^18^F-Alfatide II PET/CT scans of responding tumors (**a** SUVmax = 5.18) and non-responding tumors (**b** SUVmax = 9.80). (*Top panel*) Scans from a patient with ^18^F-Alfatide uptake at baseline and 4 weeks after CCRT who showed a partial response to CCRT, and (*bottom panel*) scans from another patient with stable disease

**Fig. 2:**
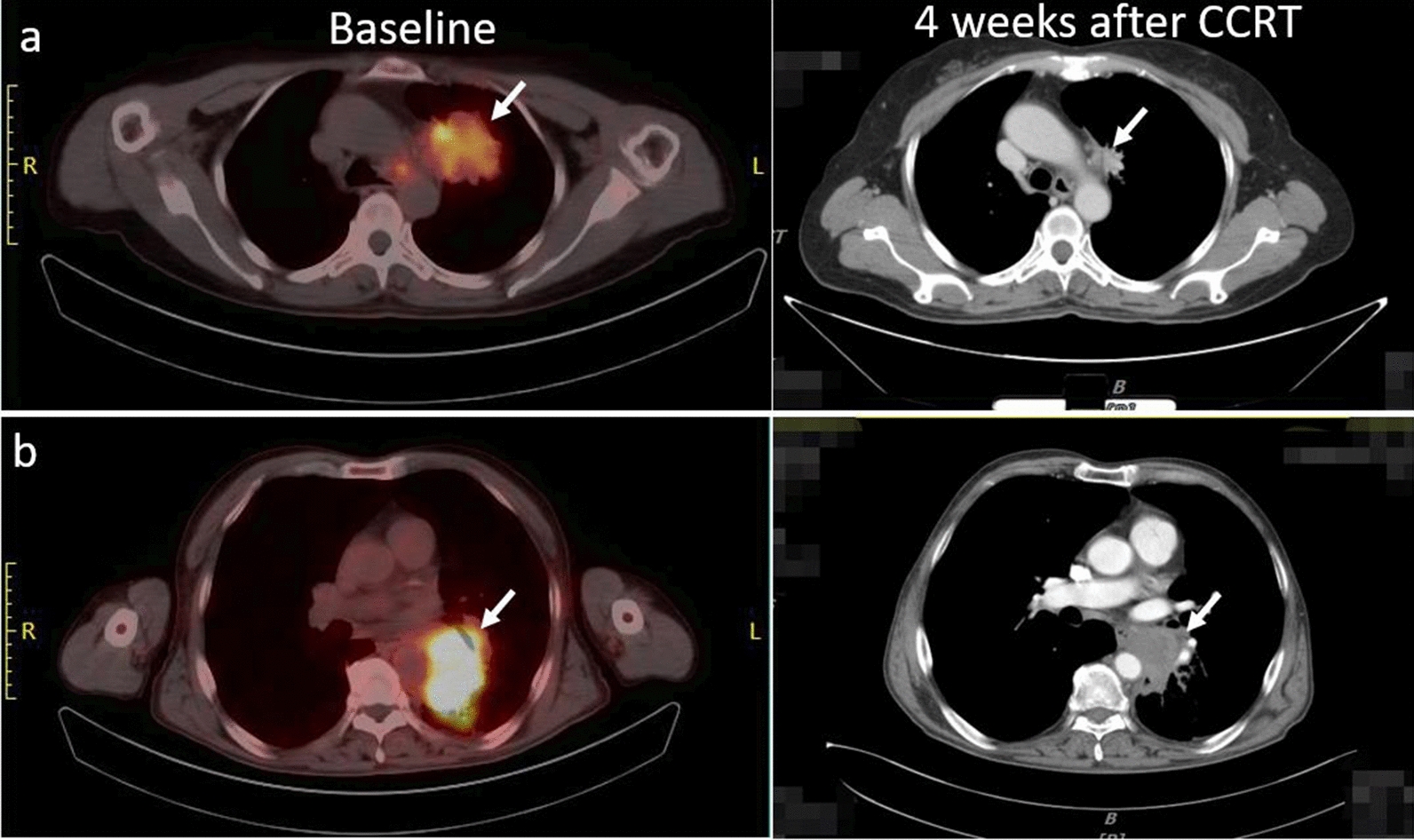
ROC curve analysis of the predictive value of SUVs on baseline ^18^F-Alfatide II PET/CT imaging for short-term response to CCRT, revealing a significant AUC of 0.764 (*P* = 0.018) for TBR_P_ (cutoff value, 6.52)

Several differences in SUV values were observed between responders and non-responders (Table 3; Fig. 1). SUV_P_ and TBR_P_ were significantly higher in non-responders than in responders (6.55 ± 2.74 vs. 4.61 ± 1.94, *P* = 0.039; 10.49 ± 7.58 vs. 7.73 ± 6.09, *P* = 0.023). ΔD was significantly greater in responders (2.78 ± 1.37) than in non-responders (-0.16 ± 1.33, *P* < 0.001). ROC curve analysis for the individual parameters, applying ‘responder’ as the dichotomous characteristic, revealed a significant area under the curve of 0.764 (*P* = 0.018) for TBR_P_ (Fig. 2; Table 4). With a cutoff value of 6.52, derived from the Youden index, the sensitivity, specificity, and accuracy for TBR_P_ were 71.43%, 78.57%, and 75.00%, respectively.

**Table 3:** Comparison of parameters among patients with different responses

Parameters	All patients	Responders	Non-responders	*P* value
SUV_P_*	5.51 ± 2.50	4.61 ± 1.94	6.55 ± 2.74	0.039
SUV_LN_	3.47 ± 1.71	3.59 ± 1.81	3.33 ± 1.65	0.699
T/LN	2.04 ± 2.30	1.42 ± 0.53	2.75 ± 3.25	0.172
TBR_P_*	7.73 ± 6.09	5.34 ± 3.03	10.49 ± 7.58	0.023
TBR_LN_	4.43 ± 2.31	4.20 ± 2.53	4.68 ± 2.11	0.589
△D*	1.41 ± 2.0	2.78 ± 1.37	-0.16 ± 1.33	< 0.001

*△D* changes of tumor diameter before and after treatment

^*^P < 0.05

**Table 4:** Area under the curve of SUV_P_, SUV_LN_, T/LN, TBR_P_ and TBR_LN_ for predicting tumor response

Variables	Area	SE^a^	Asymptotic Sig.^b^	Asymptotic 95% confidence interval	
				Lower bound	Upeper bound
SUV_P_	0.703	0.105	0.069	0.497	0.908
SUV_LN_	0.477	0.114	0.836	0.254	0.700
T/LN	0.715	0.098	0.053	0.523	0.907
TBR_P_^*^	0.764	0.091	0.018	0.586	0.943
TBR_LN_	0.585	0.111	0.447	0.367	0.803

^*^P < 0.05

^a^Under the nonparametric assumption

^b^Null hypothesis: true area = 0.5

### Predictive value of PET parameters for survival

**Fig. 3:**
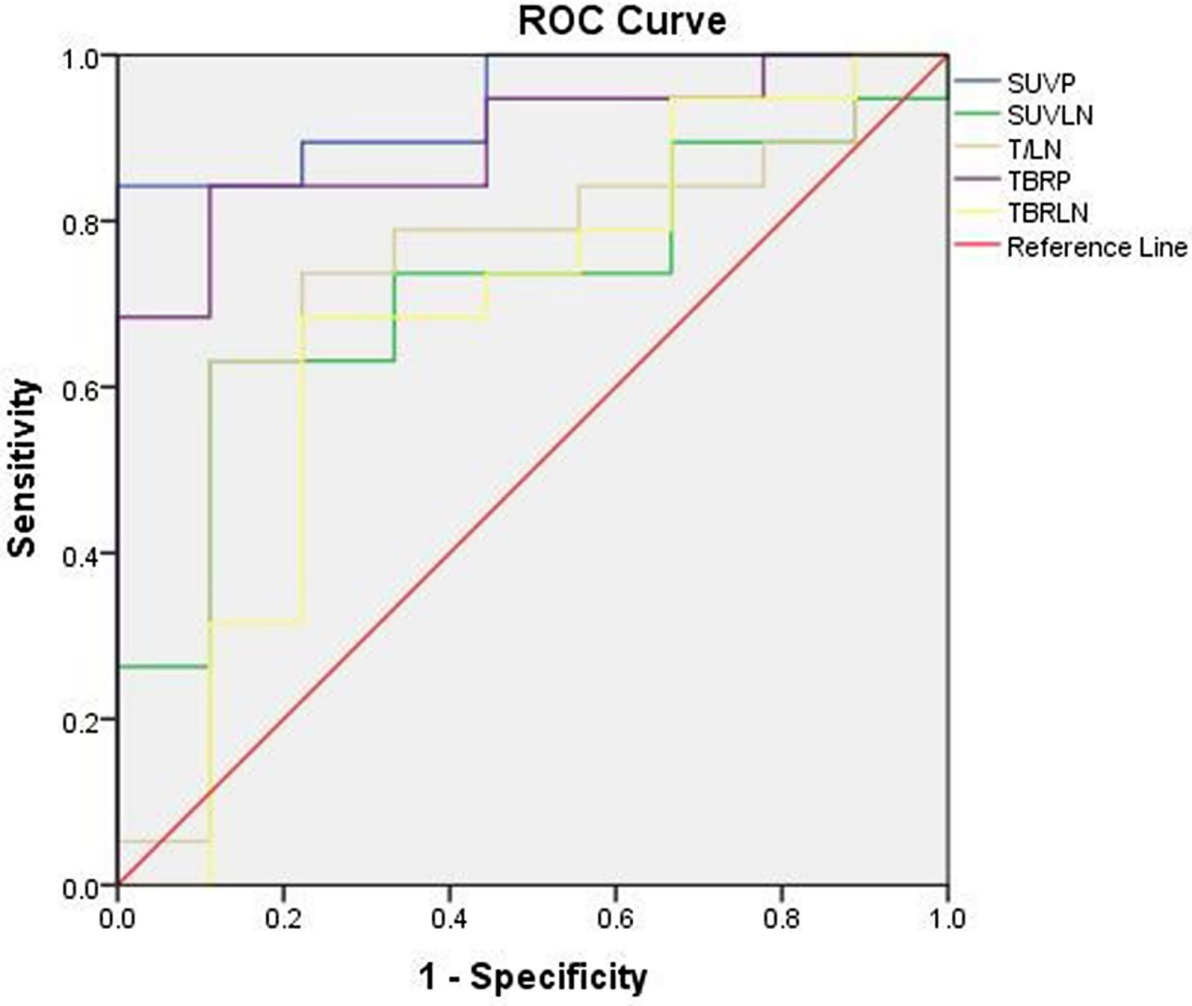
ROC curve analysis of the predictive value of SUVs on baseline ^18^F-Alfatide II PET/CT imaging, applying OS as the dichotomous characteristic, revealing significant AUC values of 0.942 (*P* < 0.001) for SUV_P_ (cutoff value, 4.64) and 0.895 (*P* = 0.001) for TBR_P_ (cutoff value, 4.92)

ROC curve analysis was performed to determine the predictive accuracy of the ^18^F-Alfatide II PET/CT parameters for survival of LA-NSCLC patients treated with CCRT. Highly significant correlations were observed between SUV_P_ and TBR_P_ and OS (*P* < 0.001 and *P* = 0.001; Fig. 3; Table 5). According to ROC curve analysis, the threshold values for SUV_P_, SUV_LN_, T/LN, TBR_P_, and TBR_LN_ were 4.64, 3.43, 1.73, 4.92, and 3.68, respectively (Table 6). The sensitivity, specificity, and accuracy of SUV_P_ for predicting OS were 84.21%, 100.00%, and 89.29%, respectively. The sensitivity, specificity, and accuracy of TBR_P_ for predicting OS were 93.75%, 66.67%, and 82.14%, respectively.

**Table 5:** Area under the curve of SUV_P_, SUV_LN_, T/LN, TBR_P_ and TBR_LN_ for predicting Survival

Variables	Area	SE^a^	Asymptotic Sig.^b^	Asymptotic 95% confidence interval	
				Lower bound	Upeper bound
SUV_P_^*^	0.942	0.042	0.000	0.860	1.000
SUV_LN_	0.719	0.099	0.065	0.524	0.914
T/LN	0.731	0.109	0.052	0.518	0.944
TBR_P_^*^	0.895	0.060	0.001	0.778	1.000
TBR_LN_	0.678	0.118	0.134	0.447	0.910

^*^P < 0.05

^a^Under the nonparametric assumption

^b^Null hypothesis: true area = 0.5

**Table 6:** The specificity, sensitivity, and accuracy of SUV_P_, SUV_LN_, T/LN, TBR_P_ and TBR_LN_ for predicting survival

Parameters	Threshold	Sensitivity (%)	Specificity (%)	Accuracy (%)
SUV_P_	4.64	84.21	100.00	89.29
SUV_LN_	3.43	92.31	53.33	71.43
T/LN	1.73	92.31	53.33	71.43
TBR_P_	4.92	93.75	66.67	82.14
TBR_LN_	3.68	86.67	53.85	71.43

## Discussion

The results of this study indicate that for LA-NSCLC patients with CCRT, the baseline values of SUV_P_, SUV_LN_, TBR_P_, TBR_LN_ and T/LN from pre-treatment ^18^F-Alfatide II PET/CT imaging correlated significantly with PFS, and SUV_P_ was negatively correlated with OS. TBR_P_ was an independent variable that may be useful for predicting short-term treatment outcome. From ROC curve analysis, SUV_P_ and TBR_P_ can predict OS with high sensitivity, specificity, and accuracy.

In clinical practice, physicians often observe differential treatment effects among different cancer patients with the same stage of disease, pathological type, and same treatment regimen [17]. The treatment of malignant tumors has entered the era of “precision medicine”. Indeed, the heterogeneity of the tumor affects the therapeutic effect and prognosis [18]. The tissues for pathological examination and gene detection are all obtained through local sampling and biopsy, and thus, it is difficult to evaluate the overall metabolism of the tumor. As new tools for the accurate diagnosis of tumors as well as prognosis prediction, new imaging methods have promoted the rapid development of individualized accurate treatment of tumors. PET is a non-invasive modality for evaluating specific molecular features and a potential tool for the prediction of treatment response. Various molecular imaging techniques have been developed for predicting tumor response to therapy, such as ^18^F-fluoro-D-glucose (FDG) PET [19], ^18^F-fluorothymidine (FLT) PET [20], ^18^F-fluroerythronitroimidazole (FETNIM) PET [21] and ^18^F-fluoromisonidazole (FMISO) PET [22].

In this study, ^18^F-Alfatide II PET was used to predict the short-term efficacy, PFS and OS of patients with LA-NSCLC, but not the effect of antiangiogenic therapy combined with radiotherapy and chemotherapy. Liu et al. reported that higher ^18^F-Alfatide II uptake on PET/CT might predict improved short-term responses and PFS after combined antiangiogenic (bevacizumab) treatments in advanced NSCLC patients [15]. Of course, at present, the standard treatment for locally advanced inoperable NSCLC is immunoconsolidation therapy after CCRT, but antiangiogenic therapy has not been recommended in the guidelines. A phase II clinical study (HELPER) explored the safety and efficacy of antiangiogenic therapy combined with CCRT for the treatment of unresectable III stage NSCLC. Although the safety was tolerable, this strategy did not prolong survival compared with radiotherapy combined with immunotherapy [23]. Ma et al. reported that continuous intravenous infusion of antiangiogenic drugs can significantly improve DFS and OS in patients with unresectable III stage NSCLC compared with intravenous administration of antiangiogenic drugs [24]. Therefore, the efficacy of antiangiogenic therapy combined with CCRT in patients with unresectable III stage NSCLC based on high RGD PET uptake is worthy of further study.

Tumor angiogenesis as evaluated by ^18^F-Alfatide II PET/CT before CCRT has been previously shown to predict the short-term treatment outcome in patients with advanced NSCLC [25], with the previous results showing that the maximum SUVs of tumor (SUVmax) and the SUVmax to SUVblood ratio (T/NTblood) were higher in non-responders (*P* = 0.0024, *P* = 0.003). The corresponding AUCs determined by ROC curve analysis were 0.815 (*P* = 0.079) and 0.889 (*P* = 0.005), respectively. These results are consistent with the results of the present study, in which we instead analyzed pre-treatment baseline data from ^18^F-Alfatide II PET/CT imaging and enrolled LA-NSCLC patients. Zhang et al. [26] also reported the predictive sensitivity of ^18^F-RGD imaging in glioblastoma patients treated with CCRT and found that both pre-treatment SUVs and intra-treatment SUVs were predictive parameters from ^18^F-RGD PET/CT images. They also found that glioblastoma patients with higher SUVs were less likely to respond to CCRT. Li Li et al. [27] reported that ^18^F-RGD uptake on PET/CT imaging pre-treatment can predict the response to antiangiogenic therapy in patients who receive apatinib therapy. They enrolled patients with lung cancer, esophageal cancer, breast cancer, cervical cancer, ovarian cancer, stomach cancer, and other cancer types who were scheduled for second- or third-line apatinib therapy and found that higher SUVs on PET/CT predicted a better response to apatinib. From these findings, we can hypothesize that the addition of anti-angiogenesis therapy in cases with a poor response to radiotherapy and chemotherapy may improve the therapeutic effect.

^18^F-FDG PET is the most widely used examination method in tumor diagnosis and staging at present, and its imaging principle is based on the characteristics of glucose metabolism within tumor cells. Its ability to predict the efficacy of NSCLC treatment has been established [28, 29]. This present study is based on the evaluation of tumor angiogenesis via ^18^F-Alfatide II PET, given that angiogenesis is closely related to the delivery of chemotherapeutic drugs and radiotherapy resistance [5]. Invasive gene detection can reveal the characteristics of gene mutation, the tumor mutation burden, programmed death ligand 1 expression, and microsatellite instability of lung cancer, which are related to the prognosis of the tumor [30]. A disadvantage is that it is difficult to obtain tumor tissue dynamically, and some lesions are too small or too close to important organs for biopsy tissue to be effectively and safely obtained. Another disadvantage is that the tissue obtained by point puncture does not represent the tumor as a whole. Through RGD PET functional imaging, dynamic, non-invasive, and three-dimensional data regarding angiogenesis within tumors can be obtained. The results of the present study demonstrate that high ^18^F-Alfatide II uptake is associated with LA-NSCLC non-response to CCRT, while previous studies have shown that high ^18^F-Alfatide II uptake is related to response to anti-angiogenic therapy for advanced NSCLC as well as PFS [15, 27]. Therefore, RGD PET may be an ideal examination method for screening the population most likely to respond to anti-angiogenic therapy.

This was a single-center study that has several limitations. First, the sample size was relatively small, and the credibility of the results needs to be further verified. Therefore, larger studies are needed, not only to verify the results but also to screen the population of LA-NSCLC patients most likely to benefit from anti-angiogenesis therapy. Additionally, the ^18^F-Alfatide II PET/CT characteristics that could predict short-term efficacy and survival were inconsistent. This may have led to inconsistent predictions, and studies with a larger sample size are needed. In this study, ^18^F-Alfatide II PET/CT imaging was performed only once in NSCLC patients before treatment, and not during or after treatment. Further research to determine whether changes in the SUVs on ^18^F-Alfatide II PET/CT correlate with prognosis is warranted. Nevertheless, these shortcomings diminish neither the potential of our findings nor the importance of dedicated prospective investigations to corroborate these findings.

## Conclusions

The results of this prospectively study confirm that pre-treatment tumor angiogenesis as evaluated by ^18^F-Alfatide II PET/CT imaging may be useful in predicting the short-term outcome of CCRT as well as the PFS and OS in patients with advanced NSCLC. For LA-NSCLC patients with higher SUVs on baseline ^18^F-Alfatide II PET/CT images, the addition of anti-vascular therapy may improve the efficacy of CCRT and prolong survival.

## Data Availability

The datasets used and/or analysed during the current study are available from the corresponding author on reasonable request.

## Abbreviations

^18^F-Alfatide: ^18^F-ALF-NOTA-PRGD2
PET/CT: Positron emission tomography /computed tomography
CCRT: Concurrent chemoradiotherapy
LA-NSCLC: Locally advanced non-small cell lung cancer
SUV_max_: Maximum uptake values
SUV_P_: Maximum uptake values of primary tumor
SUV_LN_: Maximum uptake values of metastatic lymph nodes
SUV_blood_: Mean uptake value of blood pool
TBRs: Tumor-to-background ratios
TBR_P_: Ratios of SUV_P_ to SUV_blood_, TBR_LN_, ratios of SUV_LN_ to SUV_blood_, T/LN: ratios of SUV_P_ to SUV_LN_
△D: Changes in tumor diameter before and after treatment
PFS: Progression-free survival
OS: Overall survival
CR: Complete response
PR: Partial response
SD: Stable disease
PD: Progressive disease
